# Influenza Vaccination Coverage Among Nursing Home Residents and Health Care Personnel — United States, 2024–25 Influenza Season

**DOI:** 10.15585/mmwr.mm7515a1

**Published:** 2026-04-23

**Authors:** Jeneita M. Bell, Kira Barbre, Lu Meng, Brynn Lape-Newman, Emily Wong, Austin Woods, Elizabeth J. Kalayil, Heather Dubendris, Molly Stillions Prosper, Jonathan Edwards, Minn M. Soe, David T. Kuhar, Matthew J. Stuckey, Megan C. Lindley, Andrea Benin

**Affiliations:** ^1^Division of Healthcare Quality Promotion, National Center for Emerging and Zoonotic Infectious Diseases, CDC; ^2^Chenega Government Mission Solutions, Chesapeake, Virginia; ^3^Lantana Consulting Group, East Thetford, Vermont; ^4^Chenega Enterprise Systems & Solutions, LLC, Chesapeake, Virginia; ^5^Immunization Services Division, National Center for Immunization and Respiratory Diseases, CDC.

**Summary**

**What is already known about this topic?**

Nursing home residents and health care personnel (HCP) are at increased risk for exposure to influenza, and nursing home residents are at increased risk for severe influenza. Routine annual seasonal influenza vaccination is recommended for eligible persons, including HCP and persons at increased risk for severe influenza.

**What is added by this report?**

During the 2024–25 influenza season, influenza vaccination coverage was 61.3% among nursing home residents and 42.1% among HCP working in nursing homes; coverage among HCP varied by employment type.

**What are the implications for public health practice?**

Implementation of influenza vaccination in conjunction with preventive interventions including influenza testing, antiviral treatment, proven infection prevention and control measures, and antiviral chemoprophylaxis can help protect nursing home residents and HCP from influenza illness and its complications.

## Abstract

Nursing home residents and health care personnel (HCP) are at increased risk for exposure to influenza; in addition, residents of nursing homes who acquire influenza are at increased risk for severe disease. The Advisory Committee on Immunization Practices recommends routine annual seasonal influenza vaccination for persons without contraindications, including HCP and those at increased risk for severe influenza. Nursing homes report influenza vaccination among residents and HCP to CDC’s National Healthcare Safety Network. This report describes influenza vaccination coverage among nursing home residents and HCP working in nursing homes during the 2024–25 influenza season (October 1, 2024–March 31, 2025). At the end of the 2024–25 influenza season, influenza vaccination coverage was 61.3% among nursing home residents and 42.1% among HCP who work in nursing homes; coverage among HCP varied by employment type. This study is the first comprehensive, national assessment of influenza vaccination coverage among nursing home residents and HCP who work in nursing homes in the United States. Monitoring of influenza vaccination coverage in this population at high risk for influenza exposure and severe influenza disease, along with implementation of a combination of influenza vaccination, administration of influenza antiviral medications, and other recommended practices to control the spread and severity of influenza in nursing home settings, can help protect nursing home residents and HCP against severe influenza-associated outcomes.

## Introduction

Each year, approximately 8% of the U.S. population becomes ill with influenza, a contagious respiratory illness caused by viruses that infect the nose, throat, and lungs and can lead to death. Persons who live and work in congregate settings such as nursing homes are at increased risk for exposure to influenza virus, and nursing home residents are at increased risk for complications from severe influenza (*1*). Influenza vaccination reduces the risk for severe influenza potentially leading to hospitalization (*2*) and can prevent influenza infection among nursing home residents (*3*). Increased influenza vaccination coverage among health care personnel (HCP) working in nursing homes has been associated with decreased mortality among residents (*4*). Vaccinating HCP against influenza can also reduce absenteeism among HCP (*5*), which might improve the quality of resident care by improving staffing stability (*6*). The Advisory Committee on Immunization Practices (ACIP) recommends annual seasonal influenza vaccination for persons without contraindications, including HCP and persons at increased risk for severe influenza disease (*7*). The Centers for Medicare & Medicaid Services (CMS) requires nursing homes to report influenza vaccination coverage both among nursing home residents and HCP working in nursing homes to CDC’s National Healthcare Safety Network (NHSN). This report analyzes seasonal influenza vaccination coverage among residents and HCP at nursing homes during the 2024–25 influenza season (October 1, 2024–March 31, 2025).

## Methods

### Data Source

**HCP employment categories.** Beginning with the 2022–23 influenza season, nursing homes in the United States have been required by CMS to report influenza vaccination among HCP to CDC’s NHSN at the end of each season. NHSN collects information on types of HCP employment and categorizes them into the following groups: 1) employees (all persons receiving a paycheck directly from the reporting facility); 2) licensed independent practitioners (physicians, advanced practice nurses, and physician assistants who are affiliated with the reporting facility but not directly employed by it, including postresidency fellows not on the facility’s payroll); and 3) students, trainees, or volunteers (medical, nursing, or other health professional students, interns, medical residents, or volunteers aged ≥18 years who are affiliated with but not directly employed by the facility). Employment type is determined without regard to clinical responsibility or patient contact.

**Influenza vaccination coverage among HCP.** To measure influenza vaccination coverage, facilities report an annual number of HCP working in the facility for ≥1 day during an influenza season (October 1–March 31) and the number of HCP who 1) received influenza vaccination, 2) had a medical contraindication to influenza vaccination, 3) declined vaccination, and 4) had unknown influenza vaccination status. HCP who were vaccinated outside of the nursing home facility and who provided written documentation of vaccination were categorized as vaccinated; those who reported having received a vaccination but who did not provide documentation were categorized as having an unknown vaccination status. Data collected for the 2024–25 influenza season were used in this analysis.

**Influenza vaccination coverage among nursing home residents.** Since October 2023, nursing homes have had the option to report weekly influenza vaccination coverage among nursing home residents; in January 2025, CMS began requiring nursing homes to report these data. Nursing homes report the number of residents who occupied a bed at the facility for ≥1 day during the week of data collection and the cumulative number of residents who received the current season’s influenza vaccine. Cumulative coverage data reported by nursing homes for the week of March 30, 2025, were used for analysis.

**Facility characteristics.** The following facility characteristics were obtained from the CMS provider data catalog (an official CMS open data source available to the public) and used for analysis: 1) facility ownership type (nonprofit, government, and for profit), 2) government insurance certification (Medicare, Medicaid, and dual Medicare and Medicaid), and 3) facility size tertile defined by the number of CMS-certified beds in the facility (small [fewer than 82], medium [82–120], and large [more than 120]).

### Data Analysis

**Overall resident and HCP influenza vaccination coverage.** Data reported to NHSN from CMS-certified nursing homes in all 50 U.S. states and the District of Columbia were included in this investigation. Analysis was limited to facilities that reported influenza vaccination for both residents and HCP. Pooled and facility-level influenza vaccination coverage levels were calculated for HCP and residents. Pooled influenza vaccination coverage among HCP was calculated as the total number of HCP who had received influenza vaccine, divided by the total number of HCP working in a nursing home for ≥1 day during the influenza season. HCP with a reported medical contraindication to influenza vaccination (0.89% of all HCP) were subtracted from the denominator and excluded from analysis. Pooled influenza vaccination coverage among residents was calculated as the total number of residents who received a 2024–25 influenza vaccine divided by the total number of residents living in nursing homes during the week of data collection. Facility-level influenza vaccination coverage among HCP and residents was calculated as the number of HCP or residents who received a 2024–25 influenza vaccine divided by the number of HCP or residents, respectively, in that nursing home.

**Stratified subgroup analyses.** To illustrate facility-level vaccination coverage variation, facility-level influenza vaccination coverage at the 25th, 50th, and 75th percentile distributions within each subgroup were reported. Results were further stratified by facility insurance certification (Medicaid, Medicare, or dual); ownership (nonprofit, government, or for profit); National Center for Health Statistics county-level urbanicity (large central metropolitan, large fringe metropolitan, medium metropolitan, small metropolitan, micropolitan, or noncore)*; facility size tertile; state; and U.S. region.^†^ Pooled vaccination coverage for HCP was also stratified by employment category (employee; independent licensed practitioner; and student, trainee, or volunteer). All analyses were conducted using SAS (version 9.4; SAS Institute), and maps were generated using R (version 4.4.1; R Foundation). This activity was reviewed by CDC, deemed not research, and conducted consistent with applicable federal law and CDC policy.^§^

## Results

### Influenza Vaccination Coverage Among Nursing Home Residents

Among approximately 1.2 million residents of the 13,299 nursing homes included in this analysis, the overall pooled influenza vaccination coverage was 61.3%, and the median facility-level coverage was 67.0% (IQR = 53.0%–78.6%) (Table 1). Coverage was highest among government-owned nursing homes (71.7%) and lowest among for-profit nursing homes (58.5%). In addition, coverage was highest among small facilities (65.3%) and similar among medium and large facilities (60.4%). Coverage among facilities with Medicaid insurance certification only (77%) was higher than coverage among those with both Medicare and Medicaid certification (61.3%) and those with Medicare certification only (58.7%). Regionally, coverage was highest in the Northeast (67.8%) and lowest in the Pacific region (55.5%). State-level coverage ranged from 33.0% to 80.6%, with five jurisdictions reporting influenza vaccination coverage of ≥75% among nursing home residents (North Dakota [80.6%], South Dakota [78.6%], District of Columbia [77.8%], Vermont [76.5%], and New Hampshire [75.0%]) (Figure) (Supplementary Table).

**TABLE 1:** Pooled mean influenza vaccination coverage among residents in nursing homes, by facility characteristics — National Healthcare Safety Network, United States, March 30, 2025*

Characteristic	No. of facilities	Pooled coverage			Facility-level coverage by percentile, %		
		No. of residents	No. of residents vaccinated	Coverage, % (95% CI)^†^	25th	50th	75th
**Total**	**13,299**	**1,171,380**	**718,156**	**61.3 (61.2–61.4)**	**53.0**	**67.0**	**78.6**
**Insurance certification**							
Medicare	507	25,764	15,129	58.7 (58.1–59.3)	42.2	63.6	81.8
Medicaid	111	6,689	5,151	77.0 (76.0–78.0)	67.0	80.0	91.7
Medicare and Medicaid	12,681	1,138,927	697,876	61.3 (61.2–61.4)	53.3	66.9	78.3
**Ownership**							
For profit	9,665	882,066	515,868	58.5 (58.4–58.6)	50.0	63.7	75.4
Government	801	68,749	49,320	71.7 (71.4–72.1)	62.7	75.0	84.6
Nonprofit	2,833	220,565	152,968	69.4 (69.2–69.5)	63.0	75.6	84.8
**Facility size** ^§^							
Small	4,477	215,077	140,374	65.3 (65.1–65.5)	56.7	71.0	82.4
Medium	3,885	315,419	190,602	60.4 (60.3–60.6)	51.5	65.8	77.7
Large	4,937	640,884	387,180	60.4 (60.3–60.5)	51.4	64.5	75.6
**U.S. region** *^¶^*							
Midwest	4,291	311,678	197,048	63.2 (63.1–63.4)	55.8	68.5	80.0
Mountain	493	34,448	19,492	56.6 (56.1–57.1)	48.8	63.9	75.3
Northeast	2,180	265,291	179,895	67.8 (67.6–68.0)	60.9	71.8	82.1
Pacific	1,414	119,012	66,041	55.5 (55.2–55.8)	46.4	64.8	78.1
South	4,921	440,951	255,680	58.0 (57.8–58.1)	48.1	63.6	75.9
Urbanicity**							
Large central metropolitan	2,881	310,260	179,732	57.9 (57.8–58.1)	46.2	62.6	76.4
Large fringe metropolitan	2,738	267,174	164,846	61.7 (61.5–61.9)	52.5	66.2	77.8
Medium metropolitan	2,673	243,553	146,568	60.2 (60.0–60.4)	51.1	65.1	77.5
Small metropolitan	1,401	113,533	70,714	62.3 (62.0–62.6)	54.1	67.6	79.0
Micropolitan	1,815	132,326	86,268	65.2 (64.9–65.4)	57.5	69.5	79.2
Noncore	1,791	104,534	70,028	67.0 (66.7–67.3)	61.0	72.7	82.2

* Each facility reported influenza vaccination coverage among nursing home residents each week. To approximate cumulative influenza vaccination coverage during the influenza season, coverage data reported by nursing homes for the week of March 30, 2025, were used for analysis.

^†^ 95% CIs were calculated using the mid-P method.

^§^ Facility size was determined by number of Centers for Medicare & Medicaid Services–certified beds: small (fewer than 82 beds), medium (82–120 beds), and large (more than 120 beds).

^¶^ *South:* Alabama, Arizona, Arkansas, Delaware, Florida, Georgia, Kentucky, Louisiana, Maryland, Mississippi, New Mexico, North Carolina, Oklahoma, South Carolina, Tennessee, Texas, Virginia, West Virginia, and District of Columbia; *Midwest:* Illinois, Indiana, Iowa, Kansas, Michigan, Minnesota, Missouri, Nebraska, North Dakota, Ohio, South Dakota, and Wisconsin; *Mountain:* Colorado, Idaho, Montana, Nevada, Utah, and Wyoming; *Pacific:* Alaska, California, Hawaii, Oregon, and Washington; *Northeast:* Connecticut, Maine, Massachusetts, New Hampshire, New Jersey, New York, Pennsylvania, Rhode Island, and Vermont.

** NCHS Urban-Rural Classification Scheme for Counties | National Center for Health Statistics | CDC

**FIGURE:**
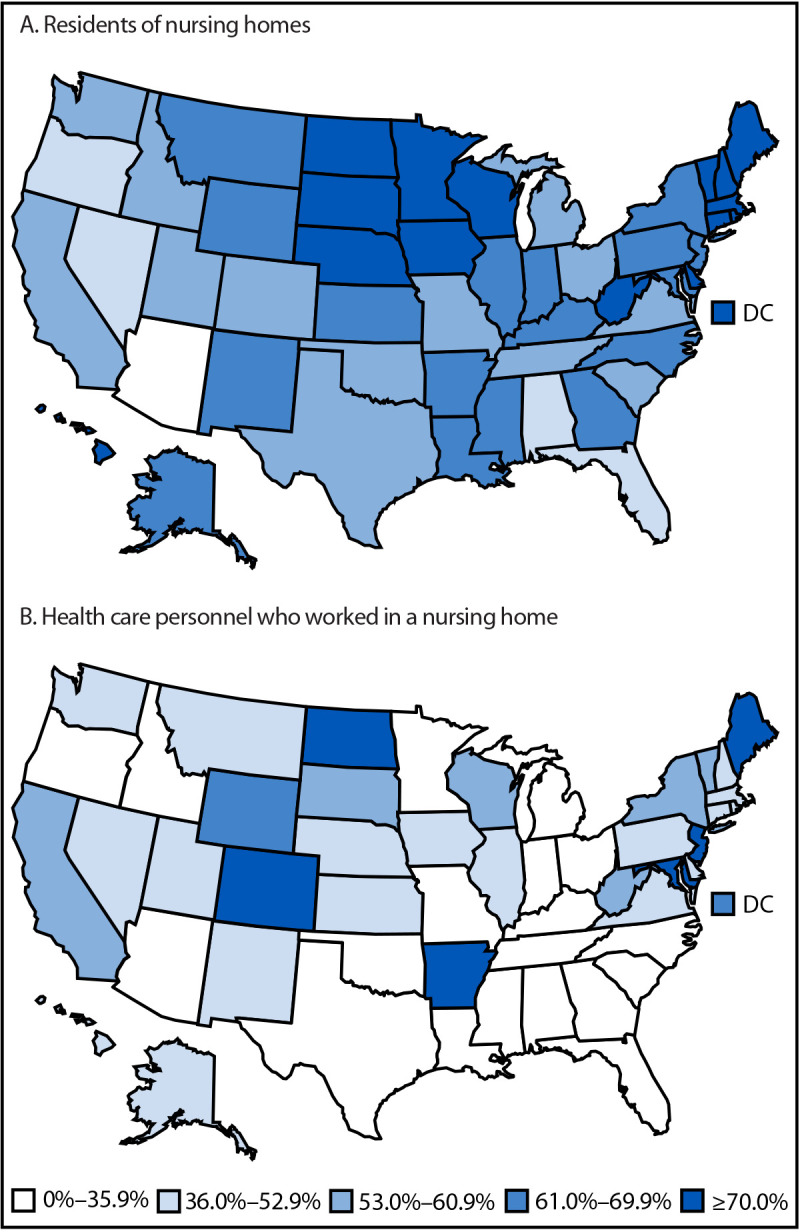
Pooled mean influenza vaccination coverage among nursing home residents (A) and health care personnel (B) — National Healthcare Safety Network, United States, 2024–25 influenza season* **Abbreviation:** DC = District of Columbia. * Each facility reported influenza vaccination coverage among nursing home residents each week. To approximate cumulative vaccination coverage over the influenza season, coverage data reported by nursing homes for the week of March 30, 2025, were used for analysis. Each facility reported summary influenza vaccination data among health care personnel who worked in the facility for ≥1 day during October 1, 2024–March 31, 2025.

### Influenza Vaccination Coverage Among HCP Working in Nursing Homes

Among approximately 2.1 million HCP working in the 13,299 nursing homes included in this analysis, the overall pooled influenza vaccination coverage was 42.1%, and the median facility-level HCP coverage was 35.1% (IQR = 16.8%–63.8%). Vaccination coverage was highest among HCP working in nonprofit nursing homes (52.6%) and lowest among those working in for-profit nursing homes (38.3%) (Table 2). In addition, influenza vaccination coverage among HCP was highest among small facilities (44.3%) and among those that accepted Medicare insurance only (54.9%). Regionally, coverage was highest in the Northeast (57.7%) and lowest in the Midwest (34.5%). Coverage was highest among students, trainees, and volunteers (55.4%) and lowest among employees (41.4%). State-level vaccination coverage ranged from 18.2% (Oklahoma) to 91.6% (Maine), with four states reporting influenza vaccination coverage of ≥75% among HCP (Maine [91.6%], New Jersey [90.7%], Arkansas [88.2%], and Colorado [78.8%]) (Figure) (Supplementary Table).

**TABLE 2:** Pooled mean influenza vaccination coverage among health care personnel working in nursing homes, by employment category and facility characteristics — National Healthcare Safety Network, United States, March 31, 2025

Characteristic	No. of facilities	Pooled coverage			Facility-level coverage by percentile, %		
		No. of HCP	No. of HCP vaccinated	Coverage, % (95% CI)^†^	25th	50th	75th
**Total**	**13,299**	**2,099,445**	**884,353**	**42.1 (42.1–42.2)**	**16.8**	**35.1**	**63.8**
**Employment category**							
Employee	13,299	1,908,559	789,599	41.4 (41.3–41.4)	NA	NA	NA
Licensed independent practitioner	13,299	97,778	43,183	44.2 (43.9–44.5)	NA	NA	NA
Student, trainee, or volunteer	13,299	93,108	51,571	55.4 (55.1–55.7)	NA	NA	NA
**Insurance certification**							
Medicare	507	77,012	42,261	54.9 (54.5–55.2)	25.9	50.6	85.2
Medicaid	111	16,403	8,720	53.2 (52.4–53.9)	25.3	59.0	83.6
Medicare and Medicaid	12,681	2,006,030	833,372	41.5 (41.5–41.6)	16.5	34.5	62.4
**Ownership**							
For profit	9,665	1,461,591	559,470	38.3 (38.2–38.4)	14.6	31.1	57.1
Government	801	140,798	63,534	45.1 (44.9–45.4)	21.8	41.9	67.7
Nonprofit	2,833	497,056	261,349	52.6 (52.4–52.7)	25.0	50.0	82.1
**Facility size** ^§^							
Small	4,477	494,762	218,983	44.3 (44.1–44.4)	18.8	37.8	67.7
Medium	3,885	565,477	222,406	39.3 (39.2–39.5)	15.3	31.8	58.3
Large	4,937	1,039,206	442,964	42.6 (42.5–42.7)	16.6	35.0	64.2
**U.S. region** *^¶^*							
Midwest	4,291	591,405	204,155	34.5 (34.4–34.6)	12.0	26.8	50.3
Mountain	493	72,302	41,117	56.9 (56.5–57.2)	31.3	57.3	85.7
Northeast	2,180	450,645	260,206	57.7 (57.6–57.9)	35.2	56.6	83.6
Pacific	1,414	229,830	117,161	51.0 (50.8–51.2)	33.7	50.5	69.6
South	4,921	755,263	261,714	34.7 (34.5–34.8)	13.1	26.8	53.8
Urbanicity**							
Large central metropolitan	2,881	535,912	231,281	43.2 (43.0–43.3)	18.1	38.6	65.5
Large fringe metropolitan	2,738	469,280	212,475	45.3 (45.1–45.4)	17.8	37.8	70.2
Medium metropolitan	2,673	447,920	178,281	39.8 (39.7–39.9)	15.8	32.1	59.3
Small metropolitan	1,401	218,260	88,616	40.6 (40.4–40.8)	16.1	32.1	59.0
Micropolitan	1,815	243,603	95,906	39.4 (39.2–39.6)	15.4	31.3	59.4
Noncore	1,791	184,470	77,794	42.2 (41.9–42.4)	17.6	35.8	67.0

**Abbreviations**: HCP = health care personnel; NA = not applicable.

* Each facility reported summary influenza vaccination data among HCP working in the facility for ≥1 day during October 1, 2024–March 31, 2025.

^†^ 95% CIs were calculated using the mid-P method.

^§^ Facility size was determined by number of Centers for Medicare & Medicaid Services–certified beds: small (fewer than 82 beds), medium (82–120 beds), and large (more than 120 beds).

*^¶^ South: *Alabama, Arizona, Arkansas, Delaware, Florida, Georgia, Kentucky, Louisiana, Maryland, Mississippi, New Mexico, North Carolina, Oklahoma, South Carolina, Tennessee, Texas, Virginia, West Virginia, and District of Columbia; *Midwest:* Illinois, Indiana, Iowa, Kansas, Michigan, Minnesota, Missouri, Nebraska, North Dakota, Ohio, South Dakota, and Wisconsin; *Mountain:* Colorado, Idaho, Montana, Nevada, Utah, and Wyoming; *Pacific:* Alaska, California, Hawaii, Oregon, and Washington; *Northeast:* Connecticut, Maine, Massachusetts, New Hampshire, New Jersey, New York, Pennsylvania, Rhode Island, and Vermont.

** NCHS Urban-Rural Classification Scheme for Counties | National Center for Health Statistics | CDC

## Discussion

During the 2024–25 influenza season, approximately three in five nursing home residents (61.3%) and two in five HCP working in nursing homes (42.1%) received an influenza vaccine. The 2024–25 season was the first during which nursing homes were required to report influenza vaccination coverage among residents. Influenza vaccination coverage among nursing home residents was lower than that among the general population aged ≥75 years during the same seasonal period (75.6%). This difference in vaccination coverage between these two populations might be partially due to differences in how the data were collected but also might indicate barriers to vaccination that are unique to nursing home residents.

Although facility-level characteristics associated with influenza vaccination varied between residents and HCP, coverage among both populations was highest in the Northeast and among small facilities. Influenza vaccination coverage among nursing home residents was highest among facilities with Medicaid certification only, which are also known as long-stay nursing homes. This finding is consistent with other reports indicating that influenza vaccination coverage is higher among residents of long-stay nursing homes than among residents of short-stay (<30 days) nursing homes. Some short-stay nursing home residents are discharged from the nursing home before vaccines are offered during the influenza season or have an unknown vaccination status (*8*). To prevent the spread of influenza in nursing homes, considering these facility-level characteristics could be beneficial during the implementation of influenza vaccination activities in conjunction with other testing, treatment, and infection prevention and control measures.

Influenza vaccination coverage among HCP working in nursing homes during the 2024–25 influenza season was lower than that during the 2023–24 influenza season (45.4%); coverage also remained substantially lower than the 80.7% reported among HCP in acute care hospitals for 2023–24 season (*9*). A recent study found that lack of confidence in the influenza vaccine among HCP in U.S. nursing homes in average- and low-coverage states was driven by concerns about potential side effects, vaccine effectiveness, and the necessity for vaccination. The same study found that influenza vaccination might be increased through interventions such as offering educational campaigns, on-site vaccination, and vaccine recommendations by trusted personnel (*10*). These findings underscore the possibility that influenza vaccination among HCP at nursing homes could be increased by implementing currently recommended strategies and tailoring approaches by employee category.

### Limitations

The findings in this report are subject to at least three limitations. First, because of differences in data collection, the calculation of influenza vaccination coverage among HCP included all HCP eligible to work in the facility at any time during the influenza season, whereas the calculation for vaccination among residents only included residents in the facility during the week of data collection. Therefore, these denominators and the resulting calculations cannot be directly compared. Second, the requirement that HCP who received influenza vaccination outside of the nursing home facility provide written documentation to be categorized as vaccinated might have resulted in underestimates of influenza vaccination coverage among HCP, especially those not directly employed by the facility. Finally, this analysis was conducted using aggregate data reported to NHSN at the facility level. Therefore, vaccination coverage could not be stratified by individual covariates that might enable an assessment of differences in coverage by factors such as age and comorbidities.

### Implications for Public Health Practice

This analysis found that during the 2024–25 influenza season, influenza vaccination coverage among HCP working in nursing homes was lower than coverage reported for the previous season. This was the first season for which comprehensive national data on influenza vaccination among residents in nursing homes were available. To prevent the spread of influenza in nursing homes, these facilities are recommended to implement influenza vaccination in combination with other practices for controlling outbreaks, such as influenza testing, proven infection prevention and control measures, antiviral treatment, and antiviral chemoprophylaxis. In addition, influenza vaccination of HCP has been associated with reduced mortality among nursing home residents (*4*) and is an important strategy for ensuring stability of staffing in nursing homes and providing optimal care to residents (*5*,*6*).

## Footnotes

* Large central metropolitan counties were located in metropolitan areas with populations of ≥1 million persons and contained the entire population of the largest principal city, were entirely contained in the largest principal city, or contained at least 250,000 residents of any principal city in the metropolitan area. Large fringe metropolitan counties were located in metropolitan areas with populations of ≥1 million persons but did not qualify as large central metropolitan counties. Medium metropolitan counties were located in metropolitan areas with populations of 250,000–999,999 persons. Small metropolitan counties were located in metropolitan areas with populations of 50,000–249,999 persons. Micropolitan counties were located in micropolitan areas containing at least one urban area with a population of 10,000–49,999 persons. Noncore counties did not meet criteria to be considered either metropolitan or micropolitan. NCHS Urban-Rural Classification Scheme for Counties | National Center for Health Statistics | CDC

† *South:* Alabama, Arizona, Arkansas, Delaware, Florida, Georgia, Kentucky, Louisiana, Maryland, Mississippi, New Mexico, North Carolina, Oklahoma, South Carolina, Tennessee, Texas, Virginia, West Virginia, and District of Columbia; *Midwest:* Illinois, Indiana, Iowa, Kansas, Michigan, Minnesota, Missouri, Nebraska, North Dakota, Ohio, South Dakota, and Wisconsin; *Mountain: *Colorado, Idaho, Montana, Nevada, Utah, and Wyoming; *Pacific:* Alaska, California, Hawaii, Oregon, and Washington; *Northeast:* Connecticut, Maine, Massachusetts, New Hampshire, New Jersey, New York, Pennsylvania, Rhode Island, and Vermont.

§ 45 C.F.R. part 46.102(l)(2), 21 C.F.R. part 56; 42 U.S.C. Sect. 241(d); 5 U.S.C. Sect. 552a; 44 U.S.C. Sect. 3501 et seq.
